# Risikofaktoren und Präventionsstrategien periprothetischer Femurfrakturen in der Hüftendoprothetik

**DOI:** 10.1007/s00132-024-04566-8

**Published:** 2024-09-24

**Authors:** Assil-Ramin Alimy, Pauline Julie Soltys, Jan Hubert, Christian Ries, Frank Timo Beil, Tim Rolvien

**Affiliations:** Klinik für Unfallchirurgie und Orthopädie, Lehrstuhl für Orthopädie, Universitätsklinikum Hamburg-Eppendorf, Martinistraße 52, 20246 Hamburg, Deutschland

**Keywords:** Knochenmineraldichte, DXA, Osteoporose, Periprothetische Fraktur, Hüfttotalendoprothese, Bone mineral density, DXA, Osteoporosis, Periprosthetic fracture, Total hip arthroplasty

## Abstract

**Hintergrund:**

Periprothetische Frakturen stellen eine bedeutende Komplikation der Endoprothetik dar, insbesondere nach Implantation einer Hüfttotalendoprothese (Hüft-TEP). Durch den demographischen Wandel wird prognostiziert, dass mit der steigenden Anzahl von Hüft-TEP-Implantationen auch eine Zunahme von periprothetischen Femurfrakturen (PPF) in den nächsten Jahrzehnten zu erwarten ist. Trotz der daraus abzuleitenden hohen klinischen Relevanz existiert bisher keine umfassende Übersicht zu Risikofaktoren und möglichen präventiven Ansätzen von PPF.

**Ziel der Arbeit:**

Ziel dieser Übersichtsarbeit ist es, die aktuellen Erkenntnisse und Daten aus verschiedenen Studien darzustellen und daraus evidenzbasierte Empfehlungen für die klinische Praxis abzuleiten.

**Material und Methoden:**

Narratives Review.

**Ergebnisse:**

Das Auftreten von PPF kann durch verschiedene Risikofaktoren wie höheres Lebensalter, weibliches Geschlecht und rheumatische Erkrankungen begünstigt werden. Bei Vorliegen dieser Risikofaktoren sollte vor der Primäroperation die Indikation zur Untersuchung der Knochengesundheit, inklusive DXA-Messung, großzügig gestellt werden.

**Schlussfolgerungen:**

Eine individualisierte Herangehensweise ist bei der Planung und Implantation einer Hüft-TEP essenziell, um das Risiko für PPF zu minimieren. Bei nachgewiesener Osteoporose oder einem Alter von ≥ 70 Jahren bei Frauen bzw. ≥ 75 Jahren bei Männern sollte eine zementierte Schaftverankerung gewählt werden, da zementfreie Schäfte in diesem Kontext mit einem erhöhten Risiko für PPF verbunden sind. Insgesamt sollte das klinische Risikoprofil bei der präoperativen Planung und der postoperativen Nachsorge berücksichtigt werden, um diese Komplikation zu reduzieren und die Patientenversorgung zu verbessern.

## Abstract (en)

**Background:**

Periprosthetic fractures represent a major complication following joint replacement surgery, particularly total hip arthroplasty (THA). Due to demographic changes, a rising number of THAs is expected to lead to an increase in periprosthetic femoral fractures (PPF) in the coming decades. Despite the resulting high clinical relevance, there is as yet no comprehensive overview of risk factors and possible preventive approaches to PPF.

**Objectives:**

The aim of this review is to present current findings and data from various studies and to derive evidence-based recommendations for clinical practice.

**Methods:**

Narrative review.

**Results:**

Various factors, such as advanced age, female sex, and rheumatic conditions, can increase the risk of PPF. In the presence of risk factors, a comprehensive assessment of bone health, including DXA osteodensitometry, should be considered prior to surgery.

**Conclusions:**

An individualized approach is essential in the planning and execution of THAs to minimize the risk of PPF. In cases of confirmed osteoporosis or for women aged ≥ 70 years and men aged ≥ 75 years, cemented stem fixation should be chosen, as uncemented stems are associated with an increased risk of PPF. Overall, the clinical risk profile should be considered in preoperative planning and postoperative care to reduce this complication and improve patient care.

Periprothetische Femurfrakturen stellen eine schwerwiegende und deutlich zunehmende Komplikation der Hüftendoprothetik dar. Eine prä- und postoperative Evaluation von Risikofaktoren sowie gegebenenfalls die Einleitung einer Osteoporosediagnostik und -therapie haben ein hohes Potenzial, die Inzidenz periprothetischer Femurfrakturen zu reduzieren. Diese Übersichtsarbeit beschreibt die aktuelle Evidenz zu Risikofaktoren und Präventionsstrategien von periprothetischen Femurfrakturen.

Der endoprothetische Gelenkersatz gehört zu den häufigsten und erfolgreichsten Operationen und führt zu kontinuierlich hohen Zufriedenheitsraten [1]. Aufgrund des demographischen Wandels ist der Bedarf an Endoprothesen in den letzten Jahren gestiegen und ein weiterer Anstieg wird prognostiziert [2]. Die Hüfttotalendoprothese (Hüft-TEP) ist mit über 175.000 Operationen im Jahr 2022 die am häufigsten durchgeführte Gelenkersatzoperation in Deutschland [3]. Mit der zunehmenden Anzahl an durchgeführten Operationen steigt auch die Wahrscheinlichkeit von Komplikationen, die in diesem Zusammenhang auftreten können.

Besonders schwerwiegende Komplikationen sind dabei periprothetische Frakturen, die in der Hüftendoprothetik laut des Jahresberichts des Endoprothesenregisters Deutschland (EPRD) für 15,9 % der Revisionsoperationen verantwortlich waren und somit nach Infektionen (22,7 %) und Prothesenlockerungen (16,4 %) den dritthäufigsten Grund für eine Revisionsoperation darstellten [3]. Im 10-Jahres-Zeitraum der EPRD-Erhebung ist der Anstieg der periprothetischen Frakturen dabei mit Abstand am stärksten. Periprothetische Frakturen lassen sich generell nach dem Zeitpunkt des Auftretens in intra- und postoperative Frakturen unterteilen [4]. Ferner kann zwischen periprothetischen Azetabulum- und Femurfrakturen unterschieden werden. Während periprothetische Frakturen des Azetabulums selten sind, spielen periprothetische Femurfrakturen mit einer Inzidenz von 1–4 % eine deutlich größere Rolle [5–8]. Daher thematisieren wir in dieser Übersichtsarbeit periprothetische Femurfrakturen nach Hüft-TEP und kürzen diese als „PPF“ ab.

PPF sind mit einer erheblichen Morbidität und einer 1‑Jahres-Mortalitätsrate von mehr als 10 % assoziiert [9–11]. Für die nächsten 30 Jahre wird eine Zunahme von PPF um durchschnittlich 4,6 % pro Jahrzehnt prognostiziert [12]. Trotz der hohen klinischen Relevanz existiert bisher keine umfassende Übersicht über PPF und deren Prävalenz, Risikofaktoren sowie mögliche präventive Ansätze. Daher stellen wir in dieser Übersichtsarbeit aktuelle Erkenntnisse und Daten verschiedener Studien dar und leiten Empfehlungen für die klinische Praxis ab.

## Risikofaktoren

In der Literatur sind verschiedene Risikofaktoren beschrieben, die das Auftreten von PPF begünstigen. Hierzu zählen unter anderem ein hohes Lebensalter, weibliches Geschlecht, inflammatorische Arthritiden wie die rheumatoide Arthritis (RA), eine Osteoporose sowie lokale, implantatassoziierte Osteolysen und Knochendefekte [13, 14].

### Alter

Ein fortgeschrittenes Lebensalter stellt einen bedeutenden Risikofaktor für PPF dar. Das durchschnittliche Lebensalter von Patienten bei Hüft-TEP-Implantation liegt laut Studien bei über 60 Jahren [15, 16]. Somit zeichnet sich dieses Kollektiv oft durch eine typische altersassoziierte Abnahme der Knochenmasse aus. Hierbei kommt es unter anderem zu einer Abnahme der mechanischen Eigenschaften des Knochens aufgrund einer geringeren Knochenmineraldichte, erhöhter Porosität und Veränderungen der Mikroarchitektur [17]. Die Bedeutung des Alters konnte unter anderem durch eine Analyse von 6458 zementierten Hüft-TEP unterstrichen werden, wobei Patienten über 70 Jahre ein 2,9fach höheres Risiko (95 %-CI: 2,0–4,3) für PPF aufwiesen. Bei Patienten über 80 Jahren war das Risiko sogar 4,4fach erhöht (95 %-CI: 2,9–6,4) im Vergleich zu Patienten unter 80 Jahren [18]. Dementsprechend sollte ein fortgeschrittenes Lebensalter bei der präoperativen Risikostratifizierung berücksichtigt werden.

### Weibliches Geschlecht

Das Geschlecht spielt im Kontext der Inzidenz von PPF ebenfalls eine Rolle. Frauen haben aufgrund von biologischen Faktoren ein erhöhtes Risiko für osteoporoseassoziierte Frakturen und dementsprechend auch für PPF. Eine retrospektive Studie mit mehr als 50.000 Patienten, die eine primäre Hüft-TEP erhalten hatten, zeigte, dass Frauen ein 1,5fach höheres Risiko für PPF hatten als Männer [19]. Dieses erhöhte Risiko betont die dringende Notwendigkeit eines umfassenden Osteoporose-Screenings und gegebenenfalls einer -Therapie bei Frauen, die sich einer Hüft-TEP-Operation unterziehen.

### Rheumatoide Arthritis

Die RA stellt aufgrund ihres systemischen und chronischen Charakters eine erhebliche Herausforderung für die Knochengesundheit dar. Die Inflammation bei der RA erhöht die Knochenfragilität und resultiert somit in einem deutlich erhöhten Risiko für PPF [20–22]. Zudem stellen Medikamente zur Behandlung der RA, wie Glukokortikoide, ein zusätzliches Risiko für das Auftreten von Frakturen dar [23].

### Osteoporose

**Abb. 1:**
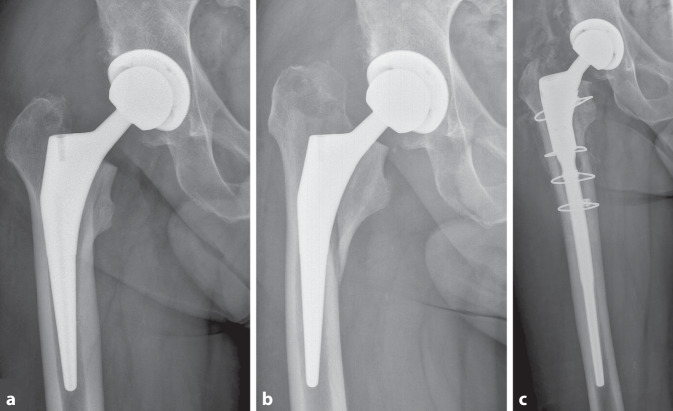
Postoperative periprothetische Fraktur. **a** Röntgenaufnahme (Hüftgelenk anteroposterior) eines 64-jährigen Patienten mit einer Osteoporose (T-Score −2,9) nach extern durchgeführter Primärimplantation einer Hüfttotalendoprothese und **b** postoperativer periprothetischer Fraktur (Vancouver B2) mit Schaftsinterung. **c** Es erfolgte die Revision mittels Cerclage-Osteosynthese und zementfreiem Langschaft

Ein wesentlicher Risikofaktor für PPF ist eine verminderte Knochenmineraldichte [13, 24, 25]. Im ersten Fallbeispiel berichten wir von einem 64-jährigen Patienten, der eine zementfreie Hüft-TEP erhalten hatte (Abb. 1a). Wenige Wochen postoperativ erlitt er nach einem Sturz aus eigener Körperhöhe eine PPF mit Schaftsinterung (Abb. 1b), welche mittels Cerclage-Osteosynthese und zementfreiem Langschaft chirurgisch adressiert wurde (Abb. 1c). Eine DXA-Messung der kontralateralen Hüfte ergab eine Osteoporose (T-Score −2,9).

Die Weltgesundheitsorganisation (WHO) definiert die Osteoporose auf der Basis von Dual-Energy X-ray Absorptiometrie(DXA)-Messungen als eine Knochenmineraldichte von weniger als ≤ −2,5 Standardabweichungen (T-Score) im Vergleich zu einer Referenzkohorte gesunder junger Frauen [26]. Ein T‑Score zwischen −1,0 und −2,5 wird als Osteopenie klassifiziert. Der Zusammenhang zwischen PPF und Osteoporose ist allein aufgrund der Tatsache zu vermuten, dass die oben beschriebenen Risikofaktoren wie fortgeschrittenes Alter, weibliches Geschlecht und rheumatoide Arthritis ebenfalls klassische Risikofaktoren der Osteoporose sind. In diesem Kontext ist hervorzuheben, dass 18 % der Hüft-TEP-Patienten in die Kategorie der Osteoporose und 41 % in die Kategorie der Osteopenie eingeordnet werden konnten [16]. Bei Patienten, bei denen bereits eine PPF eingetreten war, zeigte sich sogar eine Osteoporoseprävalenz von 45 % [27]. Aufgrund der Zunahme von PPF sowie der Behandlungslücke der Osteoporose im Kontext endoprothetischer Operationen wurden diese bereits als „Osteoporosekrise“ oder „Osteoporoseepidemie“ bezeichnet [13]. Die International Society for Clinical Densitometry empfiehlt die präoperative DXA-Knochendichtemessung bei Hüft-TEP-Patienten mit bestimmten Risikofaktoren, die im klinischen Alltag jedoch selten Anwendung findet [28]. Insgesamt bleibt festzuhalten, dass die Osteoporose und Osteopenie häufige Begleiterkrankungen bei Hüft-TEP-Patienten sind und eine Diagnostik und Therapie bei Risikopatienten durchgeführt werden sollte, um das Risiko für PPF zu reduzieren.

### Lokaler periprothetischer Knochenverlust, Prothesenlockerung

Es gibt eine Reihe von Zuständen lokalen periprothetischen Knochenverlustes, die mit PPF assoziiert sind, darunter „stress shielding“, Osteolysen und Knochendefekte. Unter „stress shielding“ versteht man eine lokale Demineralisierung des Knochens als Folge der reduzierten mechanischen Belastung, die je nach Implantat an verschiedenen Stellen des Femurs nach einer Hüft-TEP-Implantation auftreten kann [29]. Ein gewisses Maß an röntgenologisch erkennbarem „stress shielding“ ist in der Regel akzeptabel, allerdings kann ein fortgeschrittenes „stress shielding“ das Risiko einer PPF erhöhen [30]. Auch aseptische oder septische Prothesenlockerungen konnten bereits mit PPF in Verbindung gebracht werden [31]. Bereits 1982 konnten Bethea et al. feststellen, dass 75 % der Patienten mit PPF Anzeichen einer Lockerung der Femurkomponente aufwiesen [32]. Eine schwedische Registerstudie berichtete, dass 66 % der Frakturen nach einer primären Hüft-TEP-Implantation mit einer Schaftlockerung einhergingen [33]. Darüber hinaus sind aseptische oder septische Schaftlockerungen häufig mit periprothetischem Knochenverlust vergesellschaftet, was für sich genommen bereits ein zusätzliches Frakturrisiko darstellt. Insgesamt sollten routinemäßige Röntgenkontrollen durchgeführt werden, um Osteolysen, Knochendefekte und eine Implantatlockerung rechtzeitig zu erkennen und entsprechende Maßnahmen zu ergreifen, bevor es zu einer PPF kommt.

## Präoperative Risikostratifizierung

### DXA

Kürzlich veröffentlichte Studien hoben die Bedeutung der Knochenqualität bei periprothetischen Frakturen hervor und zeigten, dass der T‑Score bei Patienten mit periprothetischen Frakturen deutlich niedriger war als bei Kontrollgruppen [24, 27, 34]. Entscheidend für die Bestimmung der Knochenmineraldichte ist die DXA-Untersuchung, die als Goldstandard der Osteoporosediagnostik gilt und somit von großer Bedeutung für die Abschätzung der Knochenqualität vor einer Hüft-TEP ist. Eine DXA-Messung ist nach der neuen Leitlinie des Dachverbandes Osteologie (DVO) grundsätzlich bei allen Patienten über 70 Jahren indiziert [25]. Darüber hinaus sollte eine DXA beim Vorhandensein von bestimmten Risikokonstellationen, z. B. bei Frauen ab 50 Jahren und bei Männern ab 60 Jahren und älter, und Vorhandensein von Risikofaktoren wie früheren Fragilitätsfrakturen, proximale Femurfrakturen bei einem Elternteil, Komorbiditäten wie RA, Diabetes mellitus, neurologische Erkrankungen, Depression, Herzinsuffizienz, und bei der Einnahme von Glukokortikoiden (> 2,5 mg), Opioiden usw. vorgenommen werden.

Unter der Berücksichtigung des typischen Patientenkollektivs, welches sich einer Hüft-TEP-Implantation unterzieht, bestehen somit auch häufig Indikationen zur präoperativen Durchführung einer DXA-Messung, die essenzielle Informationen über den Knochenstatus liefern kann. Da die Prothesenstandzeiten heutzutage häufig mehrere Jahrzehnte betragen und die Patienten daher erst im Laufe der Zeit in den Indikationsbereich der DXA kommen, sollten die Risikofaktoren nicht nur präoperativ, sondern auch regelmäßig postoperativ beurteilt und ggf. eine DXA-Untersuchung veranlasst werden. Insgesamt bleibt festzuhalten, dass eine frühzeitige Risikostratifizierung entscheidend ist, um präventive Strategien zur Senkung des Risikos für PPF einzuleiten und folglich eine verbesserte Patientenversorgung zu gewährleisten.

### Radiologie

In der klinischen Praxis wird die Bestimmung der Knochenmineraldichte jedoch noch zu selten durchgeführt, was hauptsächlich darauf zurückzuführen ist, dass die DXA-Messung nicht universell verfügbar ist. Jedoch ist eine ungefähre Abschätzung der Knochenqualität über die Berücksichtigung demographischer und radiologischer Kennzeichen im Rahmen einer individuellen präoperativen Risikostratifizierung möglich. Hierbei sollten sowohl das Alter als auch das Geschlecht des Patienten berücksichtigt werden. Darüber können präoperative Röntgenaufnahmen hinsichtlich morphologischer Indizes befundet werden. Hierbei bieten der Canal Flare Index (CFI) und die Dorr-Klassifikation [35, 36] eine Möglichkeit zur Einschätzung der Knochenqualität. Der CFI wurde erstmals von Noble et al. beschrieben, um die proximale Femurgeometrie in die Formen „stovepipe“ (Ofenrohr), „normal“ und „champagne-fluted“ (Champagnerglas) zu unterteilen [37, 38]. Der CFI wird als das Verhältnis der intrakortikalen Breite des proximalen Femurs 20 mm proximal des Trochanter minor zur intrakortikalen Breite des Isthmus gemessen. Femora mit einem CFI von weniger als 3 werden als „stovepipe“, mit einem CFI von 3–4,7 als „normal“ und mit einem CFI von 4,7–6,5 als „champagne-fluted“ bezeichnet. Während ein niedriger CFI < 3 („stovepipe“, dünne Kortikalis) tendenziell ein Risiko für PPF darstellt, konnten in einer Studie frühe postoperative PPF auch mit einem höheren CFI in Verbindung gebracht werden [39]. Wenngleich hier eine dickere Kortikalis vorliegt („champagne-fluted“), kann eine übermäßige Impaktion mit konischen Raspeln eine meta-diaphysäre Fraktur begünstigen. Die proximale Femurkonfiguration kann auch anhand der Dorr-Klassifikation eingeteilt werde, welche drei Typen (A–C) unterscheidet [36]. Der Dorr-Typ A zeigt dicke und ausgeprägte Kortizes auf AP- und lateralen Röntgenbildern, die einen engen diaphysären Kanal und eine „Trichterform“ des proximalen Femurs bilden. Typ B weist auf einen Knochenverlust in der medialen und posterioren Kortikalis hin, der zu einem breiteren diaphysären Kanal führt und bei Männern häufiger vorkommt als bei Frauen. Typ C zeigt einen erheblichen Verlust der medialen und posterioren Kortikalis mit einem relativ weiten Markraum, welcher bei älteren und inaktiven Patienten vorkommt [40]. Insgesamt konnte gezeigt werden, dass eine zementfreie Verankerung bei einer Femurmorphologie vom Typ Dorr C mit PPF assoziiert ist [41]. Insbesondere bei einer Femurkonfiguration vom Typ C sollte folglich eine zementierte Verankerung bevorzugt verwendet werden.

### Chirurgische Implikationen

**Abb. 2:**
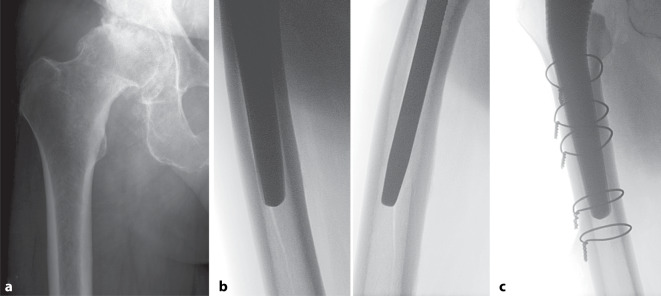
Intraoperative periprothetische Fraktur. **a** Präoperative Röntgenaufnahme (Hüftgelenk anteroposterior) eines 64-jährigen Patienten mit einer Osteopenie und Dorr-Typ B Femur (T-score −2,0). **b** Intraoperative Durchleuchtung mit Darstellung der periprothetischen Fraktur und **c** der Osteosynthese mittels Cerclagen

Die Erkenntnisse aus der erfolgten Risikostratifizierung können eine zentrale Rolle in der präoperativen Planung und chirurgischen Vorgehensweise bei der Implantation einer Hüft-TEP spielen. Eine wesentliche Komponente ist hierbei die Art der femoralen Implantatverankerung. Insbesondere vor dem Hintergrund möglicher Komplikationen herrscht noch heute eine anhaltende Debatte, ob bei der primären Hüft-TEP-Implantation und Vorliegen von bestimmten Risikofaktoren eine zementfreie oder zementierte Schaftverankerung gewählt werden sollten. Im zweiten Fallbeispiel zeigen wir einen 64-jährigeren Patienten mit primärer Koxarthrose und Osteopenie (T-Score −2,0) (Abb. 2a), bei dem bei der Implantation einer zementfreien Hüft-TEP eine intraoperative PPF aufgetreten ist (Abb. 2b), welche mittels Cerclage-Osteosynthese und zementfreiem Schaftwechsel behandelt wurde (Abb. 2c).

Die Konsequenz einer niedrigen Knochenmineraldichte für das operative Vorgehen, insbesondere der Schaftverankerung, wird immer wieder diskutiert. So gaben insgesamt 60 % der befragten Orthopäden in einer früheren Umfrage an, dass sie bei niedriger Knochenmineraldichte eine zementierte Schaftverankerung bevorzugen würden [42]. Eine biomechanische Studie konnte zeigen, dass eine zementfreie Schaftverankerung bei Patienten mit reduzierter Knochenqualität ein höheres Risiko für PPF darstellt, während die Zementierung über eine interne Versteifung des Markraums insgesamt protektiv wirkte [43]. Verschiedene Studien haben sich genauer mit dieser Frage aus klinischer Sicht beschäftigt. Eine Registerstudie zeigte, dass PPF bei 0,07 % der zementierten Schäfte und 0,47 % der zementfreien Schäfte auftraten, wobei zementfreie Schäfte ein höheres Risiko für diese Frakturen aufwiesen, mit einem relativen Risiko (RR) von 8,72 (95 %-CI, 7,37–10,32) [44]. Eine weitere Studie konnte zeigen, dass Frauen über 75 Jahren, die zementfrei versorgt wurden, ein erhöhtes Risiko für frühzeitige PPF aufwiesen [45]. Dies spiegelt sich ebenfalls in den Daten des EPRD aus dem Jahresbericht 2023 wider, wo ein allgemein höheres Ausfallrisiko bei Patienten über 75 Jahren mit zementfreier Schaftversorgung festgestellt wurde [3]. Dies konnte ebenfalls in einer Untersuchung an mehr als 32.000 primären Hüft-TEP bestätigt werden, die das Risiko für intra- und postoperative Frakturen differenziert untersuchten [46]. Hier konnte festgestellt werden, dass intraoperative Frakturen 14-mal häufiger und postoperative Frakturen 10-mal häufiger bei zementfreien Schäften auftreten. Das höchste Risiko im Allgemeinen bestand bei weiblichen Patienten, die älter als 65 Jahre sind [46]. Analysen von Registerdaten aus verschiedenen Ländern zeigten ebenfalls, dass ältere Patienten bei zementfreier Versorgung ein erhöhtes Risiko für Revisionsoperationen hatten [47, 48]. Somit wird insgesamt die Bedeutung des erhöhten Lebensalters in Kombination mit der zementfreien Versorgung bei der Entstehung von PPF nochmals deutlich unterstrichen. Zusammenfassend sollte eine zementierte Schaftverankerung vorwiegend im höheren Lebensalter und bei vorliegender Osteoporose angewendet werden.

### Pharmakologische Implikationen

Die medikamentöse Behandlung der Osteoporose ist generell die Methode der Wahl, um die Knochenmineraldichte zu erhöhen und das Frakturrisiko zu senken. Angesichts der hohen Prävalenz von Osteoporose und Osteopenie bei Patienten, die sich einer Hüft-TEP-Implantation unterziehen, kann eine spezifische Osteoporosetherapie bei einer Vielzahl an Patienten eine entscheidende Rolle spielen. Verschiedene Studien konnten zeigen, dass Bisphosphonate mit einem reduzierten Risiko für Revisionsoperationen assoziiert sind [49–51], indem sie den Osteoklasten vermittelten periprothetischen Knochenverlust reduzieren [52]. Eine Registerstudie aus Großbritannien zeigte, dass Bisphosphonate mit einer fast zweifachen Verlängerung der Standzeit verbunden sind [49]. Weiterhin können antiresorptive Medikamente einen positiven Effekt auf die Überlebensdauer von Implantaten nach Gelenkersatzoperationen haben, indem sie das „stress shielding“ unabhängig von der Implantatverankerung verringern [53]. Mehrere Studien konnten zeigen, dass die periprothetische Knochenmineraldichte durch antiresorptive Medikamente wie Bisphosphonate oder Denosumab verbessert wird, was eine Verringerung des PPF-Risikos vermuten lässt [54, 55]. Dennoch ist die Verringerung des PPF-Risikos durch knochenspezifische Medikamente noch nicht ausreichend untersucht worden.

**Abb. 3:**
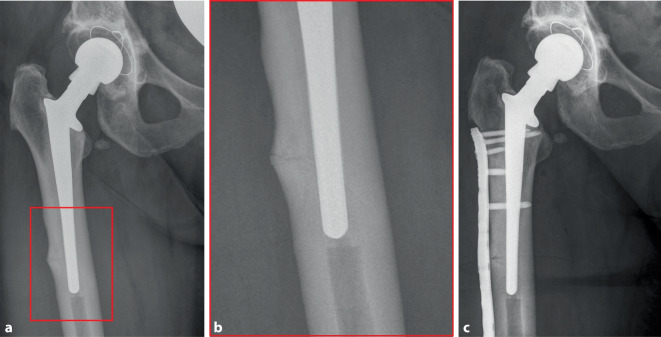
Atypische periprothetische Fraktur. **a** Röntgenaufnahme (Hüftgelenk anteroposterior) einer atypischen periprothetischen Fraktur bei einer 79-jährigen Patientin bei Zustand nach metastasiertem Mammakarzinom und 10-jähriger antiresorptiver Therapie mittels Bisphosphonaten und Denosumab. **b** Der Ausschnitt zeigt den charakteristischen Nachweis einer lateralen periostalen Verdickung und der querverlaufenden Frakturlinie. **c** Postoperatives Röntgen nach Plattenosteosynthese

Demgegenüber existieren auch Beobachtungen, dass Bisphosphonate das Risiko von PPF erhöhen können [56, 57]. Allerdings wiesen beide genannten Studien erhebliche methodische Defizite auf, da sie nicht für den Schweregrad der Osteoporose bzw. den T‑Score adjustieren, weshalb anzunehmen ist, dass der Schweregrad der Osteoporose und nicht die Bisphosphonattherapie selbst ein Risikofaktor für PPF darstellt [58]. Dennoch ist davon auszugehen, dass die langjährige Gabe von Bisphosphonaten und Denosumab in seltenen Fällen mit atypischen PPF assoziiert ist [59, 60]. In einer früheren Studie betrug die Prävalenz der atypischen PPF unter allen PFF 8,3 % [59]. Atypische PPF haben typische radiologische Kennzeichen von atypischen Femurfrakturen, darunter eine querverlaufende Frakturmorphologie, eine periostale Verdickung sowie eine unikortikale Fraktur [59]. Diesbezüglich verweisen wir auf das dritte Fallbeispiel einer 79-jährigen Patientin bei Zustand nach metastasiertem Mammakarzinom und 10-jähriger antiresorptiver Therapie mit Bisphosphonaten und Denosumab (Abb. 3a–c).

Obwohl atypische PPF selten sind, ist es wichtig, dieses Risiko zu berücksichtigen. Weitere Studien sind notwendig, um den Zusammenhang zwischen antiresorptiven Medikamenten und PPF besser zu verstehen und um die optimale Verwendung, insbesondere die Therapiedauer, im Allgemeinen zu bestimmen. Stand heute überwiegen jedoch die Vorteile der Bisphosphonattherapie, insbesondere im Hinblick auf die Verringerung des Frakturrisikos und die Verlängerung der Standzeit von Implantaten.

Insgesamt bleibt festzuhalten, dass die medikamentöse Verbesserung des lokalen Knochenstatus im Rahmen einer Hüft-TEP-Implantation und insbesondere die Wirksamkeit osteoanaboler Medikamente sowie die Verringerung des Frakturrisikos durch knochenspezifische Medikamente allgemein noch unzureichend erforscht sind. Daher sind weitere Studien notwendig, um evidenzbasierte Therapieempfehlungen formulieren zu können.

**Abb. 4:**
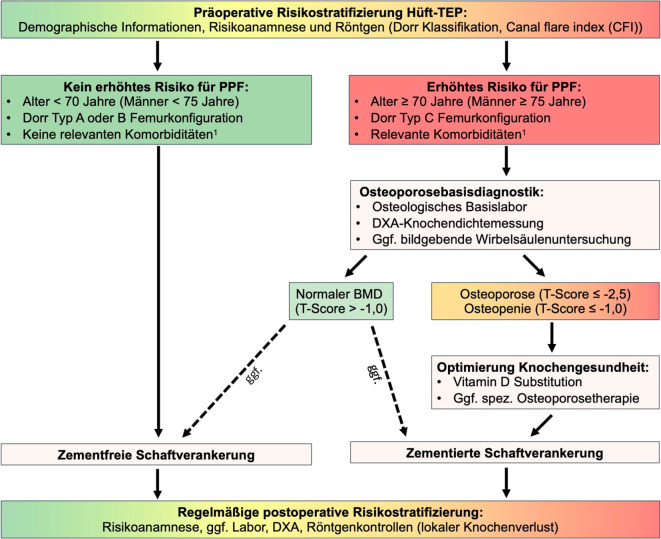
Handlungsempfehlungen für die klinische Praxis. ^1^ siehe DVO-Leitlinie Osteoporose 2023 [25]. Abkürzungen: *BMD* Knochenmineraldichte, *DXA* Dual-Energy X-ray Absorptiometrie, *HTEP* Hüfttotalendoprothese

Eine Übersicht unserer Handlungsempfehlungen findet sich in Abb. 4**.**

## Fazit für die Praxis

- Bei Vorhandensein von Risikofaktoren wie Fragilitätsfrakturen, Diabetes mellitus, rheumatoider Arthritis oder Steroidmedikation sollte die Indikation zur präoperativen Untersuchung der Knochengesundheit mittels DXA und laborchemischer Untersuchung großzügig gestellt werden.
- Dies gilt für Frauen und Männern ≥ 70 Jahren auch ohne zusätzliche Risikofaktoren.
- Bei Osteoporose (T-Score ≤ −2,5) oder Alter ≥ 70 Jahre bei Frauen bzw. ≥ 75 Jahre bei Männern sollte eine zementierte Schaftverankerung gewählt werden, da die Implantation zementfreier Schäfte mit einem erhöhten Risiko für periprothetische Frakturen verbunden ist.
- Knochenspezifische Medikamente wie Bisphosphonate oder Denosumab erhöhen die periprothetische BMD, allerdings ist die Wirksamkeit in Bezug auf die Vermeidung von periprothetischen Frakturen noch unzureichend untersucht.
- Auch postoperativ sind regelmäßige radiologische Kontrollen (lokaler Knochenverlust, Lockerung?) und eine kontinuierliche Risikostratifizierung empfohlen.

## Abkürzungen

BMD: Knochenmineraldichte
CFI: Canal Flare Index
DXA: Dual-Energy X-ray Absorptiometrie
DVO: Dachverband Osteologie
EPRD: Endoprothesenregister Deutschland
Hüft-TEP: Hüfttotalendoprothese
PPF: Periprothetische Femurfraktur
RA: Rheumatoide Arthritis
